# Allosteric Inhibition of Serotonin 5-HT_7_ Receptors by Zinc Ions

**DOI:** 10.1007/s12035-017-0536-0

**Published:** 2017-04-28

**Authors:** Grzegorz Satała, Beata Duszyńska, Tomasz Lenda, Gabriel Nowak, Andrzej J. Bojarski

**Affiliations:** Polish Academy of Sciences, Smetna 12, 31-343 Kraków, Poland

**Keywords:** Zn, Serotonin 5-HT7 receptor, Allosteric modulation, Radioligand binding

## Abstract

The allosteric regulation of G protein-coupled receptors (GPCRs) is a well-known phenomenon, but there are only a few examples of allosteric modulation within the metabotropic serotonergic receptor family. Recently, we described zinc non-competitive interactions toward agonist binding at serotonin 5-HT_1A_ receptors, in which biphasic effects, involving potentiation at sub-micromolar concentrations (10 μM) and inhibition at sub-millimolar concentrations (500 μM) of Zn^2+^ in radioligand binding assays, were consistent with both the agonist and antagonist-like effects of zinc ions observed in in vivo studies. Here, we showed new data demonstrating zinc allosteric inhibition of both agonist and antagonist binding at human recombinant 5-HT_7_ receptors stably expressed in HEK293 cells as observed by radioligand binding studies as well as zinc neutral antagonism displayed by the concentration of 10 μM in the functional LANCE assay. The allosteric nature of the effect of Zn on 5-HT_7_ receptors was confirmed (1) in saturation studies in which zinc inhibited the binding of potent orthosteric 5-HT_7_ receptor radioligands, the agonist [^3^H]5-CT, and the two antagonists [^3^H]SB-269970 and [^3^H]mesulergine, showing ceiling effect and differences in the magnitude of negative cooperativity (*α* = 0.15, 0.06, and 0.25, respectively); (2) in competition experiments in which 500 μM of zinc inhibited all radioligand displacements by non-labeled orthosteric ligands (5-CT, SB-269970, and clozapine), and the most significant reduction in affinity was observed for the 5-CT agonist (4.9–16.7-fold) compared with both antagonists (1.4–3.9-fold); and (3) in kinetic experiments in which 500 μM zinc increased the dissociation rate constants for [^3^H]5-CT and [^3^H]mesulergine but not for [^3^H]SB-269970. Additionally, in the functional LANCE test using the constitutively active HEK293 cell line expressing the 5-HT_7_ receptor, 10 μM zinc had features of neutral antagonism and increased the EC_50_ value of the 5-CT agonist by a factor of 3.2. Overall, these results showed that zinc can act as a negative allosteric inhibitor of 5-HT_7_ receptors. Given that the inhibiting effects of low concentrations of zinc in the functional assay represent the most likely direction of zinc activity under physiological conditions, among numerous zinc-regulated proteins, the 5-HT_7_ receptor can be considered a serotonergic target for zinc modulation in the CNS.

## Introduction

Identification of numerous synthetic small-molecule compounds which do not bind to the site of endogenous ligand (orthosteric binding site) but interact with other, topographically distinct, allosteric location has a range of implications for drug discovery and understanding of basic biological processes. The phenomenon of allostery, which was originally recognized more than 50 years ago as a mechanism of enzyme regulation, is currently attributed to other classes of proteins, including the G protein-coupled receptor (GPCR) superfamily [1–4]. Indeed, GPCRs have an inherent allosteric nature since the G proteins alter receptor conformation in an allosteric manner to modulate the binding and/or signaling properties of orthosteric ligands [1–4]. In addition to the discovery of new exogenous modulators, the prevalent presence of allosteric sites in GPCRs suggests that they may also serve as an interaction site for endogenous allosteric ligands [5].

The group of known allosteric GPCR modulators that function endogenously includes a variety of ions, lipids, amino acids, and peptides. Among them, the action of zinc (Zn), which is most often described at ionotropic NMDA-type glutamate receptors, has also been identified as an allosteric modulation of many GPCRs, i.e., different types of dopaminergic, adrenergic, melanocortin, opioid, and serotonin receptors [5–8]. However, regarding direct zinc interactions at specific GPCRs, diversified and frequently complex effects of Zn have been observed. At the dopamine D_1_, D_2_, and D_4_ receptors, Zn inhibits the binding of orthosteric ligands in a manner that is consistent with negative allosteric modulation [8–10]. In turn, either a negative allosteric modulation of antagonist binding or a positive allosteric modulation of agonist (epinephrine) binding has been observed for α_1A_ adrenergic receptors in contrast to functional experiments in which Zn insurmountably antagonized epinephrine activation [11]. Moreover, interactions of Zn with high-affinity sites of β_2_ adrenergic receptors enhanced the agonist affinity and agonist-stimulated cAMP accumulation, whereas interactions with a low-affinity site inhibited antagonist binding and slowed antagonist dissociation [12, 13]. The influence of Zn on melanocortin MC_1_ and MC_4_ receptor signaling has been described in the following three distinct ways: direct agonism, positive allosteric modulation of the endogenous agonist interaction, and inhibition of the endogenous inverse agonist binding [14, 15]. Zinc also inhibited orthosteric ligand binding at μ opioid receptors, whereas the δ and κ receptors were relatively insensitive to its influence [16–18]. A recent report demonstrated an unexpected role of zinc at GPR39, a member of the ghrelin peptide receptor family. In addition to activation of GPR39, which is described as a Zn^2+^-sensing receptor, zinc also allosterically enhanced intracellular signaling induced by two novel small-molecule agonists of GPR39: LY2784544 and GSK2636771 [19].

In the group of metabotropic serotonin (5-HT) receptors, the allosteric modulation of zinc ions has been demonstrated only for the 5-HT_1A_ subtype [6, 7]. Initially, by investigating native rat brain cortical membranes, Zn^2+^ was shown to function as a negative allosteric modulator of orthosteric ligand binding to 5-HT_1A_ receptors [6]. However, a recent study using HEK293 cells expressing the 5-HT_1A_ receptor revealed a biphasic effect of Zn^2+^ interactions at the 5-HT_1A_ receptor, which involved the allosteric potentiation of agonist binding at sub-micromolar Zn^2+^ concentrations and inhibition at sub-millimolar Zn^2+^ concentrations [7]. Moreover, these in vitro results were consistent with in vivo data, which indicated both the agonist and antagonist-like effects of Zn^2+^ at the 5-HT_1A_ receptor, and this dual mechanism of Zn^2+^ activity has been proposed to underlie its antidepressant-like effects observed in behavioral tests [7].

Since both zinc and brain serotonergic receptors are known to be involved in many central nervous system (CNS) functions and pathologies [20–24], it was of interest to assess whether direct interactions between Zn^2+^ and any subtype other than 5-HT_1A_ could be recognized and quantified using radioligand binding experiments and functional assays. Therefore, in this study, we focused on 5-HT_7_ receptors, which are highly co-expressed with 5-HT_1A_ receptors in the majority of brain structures and, similarly to zinc ions, have been reported to be an important for the treatment of depression [25–31].

## Materials and Methods

### Drugs

[^3^H]5-CT (135.2 Ci/mmol), [^3^H]SB-269970 (45.8 Ci/mmol), and [^3^H]mesulergine (83.1 Ci/mmol) were purchased from PerkinElmer (Waltham, USA); 5-CT, mesulergine, clozapine, and ZnCl_2_ were obtained from Sigma-Aldrich (Saint Louis, USA); and SB-269970 was obtained from Tocris (Bristol, UK).

### Expression of the Human 5-HT_7b_R Gene

The full-length human 5-HT_7b_ receptor complementary DNA (cDNA), which was cloned into the mammalian expression vector pcDNA3.1(+), was obtained from the Missouri S&T cDNA Resource Center (Bloomsburg, USA, www.cdna.org). The receptor cDNA was stably transfected into human embryonic kidney cells (HEK293 cells purchased from the American Type Culture Collection, Manassas, VA, USA) using Lipofectamine 2000 (Invitrogen, Carlsbad, USA). A clone yielding a high expression level of the 5-HT_7b_ receptor was selected during the preliminary experiments, including RT-PCR and Western blot analysis.

### Cell Culture

HEK293 cells stably expressing the human 5-HT_7b_ receptor were grown in Dulbecco’s Modified Eagle’s Medium (DMEM) (Lonza, Basel, Switzerland) supplemented with 10% dialyzed fetal bovine serum (FBS) (Lonza) and 500 μg/ml G418 sulfate (Sigma-Aldrich) at 37 °C in a humidified atmosphere with 5% CO_2_. For the membrane preparations, the cells were sub-cultured in 150-cm^2^ flasks, grown to 90% confluence, washed twice with phosphate-buffered saline (PBS) pre-warmed to 37 °C, and pelleted by centrifugation (200×*g* for 5 min) in PBS containing 0.1 mM ethylenediaminetetraacetic acid (EDTA) and 1 mM dithiothreitol (DTT). Prior to the membrane preparation, the pellets were frozen at −80 °C. For the functional experiments, the cells were sub-cultured in 75-cm^2^ flasks, grown to 90% confluence, washed twice with PBS pre-warmed to 37 °C, and pelleted by centrifugation (180×*g*) in PBS containing 0.5 mM EDTA. The supernatant was aspirated, and the cell pellet was resuspended in stimulation buffer (1× HBSS, 5 mM HEPES, 0.5 mM IBMX, 0.1% BSA).

### Cell Membrane Preparation for Radioligand Binding Experiments

Cell pellets were thawed and homogenized in 10 volumes of 50 mM Tris–HCl buffer (pH 7.7) containing 0.1 mM EDTA, using an Ultra Turrax tissue homogenizer. The pellets were centrifuged twice at 35,000×*g* for 20 min at 4 °C, with 15-min incubation at 37 °C between centrifugations. The pellets were then resuspended in the incubation buffer as described below for the radioligand binding assays. The membrane protein concentrations were determined using the Pierce™ Coomassie (Bradford) Protein Assay Kit (Thermo Fisher Scientific, Waltham, USA), with bovine serum albumin (BSA) as the standard.

### Saturation and Competition Binding Assays

Binding assays were performed using the 5-HT_7_ receptor agonist [^3^H]5-CT (0.1–8 nM for saturation or 0.8 nM for competition) and the two 5-HT_7_ receptor antagonists [^3^H]SB-269970 (0.1–16 nM for saturation or 2.5 nM for competition) and [^3^H]mesulergine (0.2–25 nM for saturation or 10 nM for competition) by incubating 5 μg of protein of the membrane suspension for 60 min at 37 °C in 96-well microtiter plates in a final volume of 200 μl. The dissociation constant of radioligand (*K*_D_), the maximal number of binding sites (B_max_), and the apparent dissociation constant of radioligand observed in the presence of modulator (*K*_App_) were measured by saturation binding experiments in the absence and presence of seven concentrations of Zn^2+^ ranging from 0.01 to 5 mM. Competition experiments with orthosteric 5-HT_7_ receptor ligands (5-CT, SB-269970, and clozapine) which were used at seven increasing concentrations from 0.1 nM to 100 μM (excluding homologous binding of [^3^H]5-CT, for which the concentration of 5-CT ranged from 0.01 nM to 10 μM) were performed in the absence and presence of Zn^2+^ at two fixed concentrations (10 and 500 μM). Non-specific binding was defined by the binding obtained in the presence of 10 μM 5-HT. The incubation buffer consisted of 50 mM Tris–HCl (pH 7.7), 4 mM MgCl_2_, 10 μM pargyline, and 0.1% ascorbic acid. The binding reactions were stopped by filtration through GF/B UniFilter plates using a harvester (PerkinElmer). The plate filters were then dried, and 20 μl of Ultima Gold MV (PerkinElmer) was added. Radioactivity was measured using a MicroBeta TriLux counter (PerkinElmer). In all experiments, the total radioligand bound never exceeded more than 10% of the total radioligand added, and thus, depletion did not affect the binding parameter measurements.

### Dissociation Assays

Dissociation rate kinetic assays were performed at 37 °C using the same buffer conditions as described for the equilibrium binding assays and 0.8 nM [^3^H]5-CT, 2.5 nM [^3^H]SB-269970, and 10 nM [^3^H]mesulergine. Non-specific binding was defined by the addition of 10 μM 5-HT. The membranes were incubated with radioligand for 60 min to achieve equilibrium. Next, 5-HT was added at a fixed concentration (10 μM) or with 10 or 500 μM of ZnCl_2_. The specifically bound radioligand was measured after different incubation durations (from 0 to 60 min), which were terminated by rapid filtration.

### Functional Evaluation: Cyclic AMP Assay

The functional properties of the compounds (5-CT, SB-269970, mesulergine, and zinc) were evaluated in the same HEK293 cells overexpressing the human 5-HT_7b_ receptor as used in the radioligand binding assays, based on their ability to increase cAMP production for agonist or inhibit 10 nM of 5-CT (EC_90_—concentration producing 90% of the maximum agonist activation) for antagonist. Additionally, the effects of SB-269970, mesulergine, and Zn^2+^ on the level of constitutive activity present in our recombinant 5-HT_7_ receptor system were also measured.

Each compound was tested at eight concentrations ranging from 0.01 nM to 100 μM. The total level of cAMP was measured using the LANCE cAMP detection kit (PerkinElmer) according to the manufacturer’s recommendations. To quantify the cAMP levels, 200 or 1000 (for measuring constitutive activity) cells/well (5 μl) were incubated with compounds (5 μl) for 30 min at room temperature in a 384-well white opaque microtiter plate. After incubation, 10 μl of the working solution (5 μl Eu-cAMP and 5 μl ULight-anti-cAMP) was added to induce cell lysis and terminate the reaction. The assay plate was incubated for 1 h at room temperature. The time-resolved fluorescence resonance energy transfer (TR-FRET) signal was detected using an Infinite M1000 PRO (Tecan, Männedorf, Switzerland) with instrument settings from the LANCE cAMP detection kit manual.

### Lactate Dehydrogenase Cytotoxicity Assay

To estimate potential ZnCl_2_-induced cell death, a colorimetric method was applied in which the amount of formazan salt that had formed after the conversion of lactate to pyruvate followed by the reduction of tetrazolium salt was proportional to the lactate dehydrogenase (LDH) activity in the sample. All procedures were conducted according to the manufacturer’s protocol (Pierce LDH Cytotoxicity Assay Kit, Thermo Fisher Scientific, Waltham, Massachusetts, USA). In brief, the level of LDH released from damaged cells into the culture medium was measured after a 30-min exposure to different concentrations of ZnCl_2_ in 96-well plates. Cell-free culture supernatants were collected from each well and incubated with the appropriate substrate mix at RT for 30 min. The intensity of the red color formed in the assay and measured at a wavelength of 490 nm was proportional to the LDH activity and the number of damaged cells. The data were normalized to the activity of LDH released from lysis buffer-treated cells (100%) and expressed as a percent of the total ± SEM from three separate experiments.

### Cell Viability Assay

The reduction of tetrazolium salts is widely accepted as a reliable method to examine cell proliferation. The yellow tetrazolium 3-(4,5-dimethylthiazolyl-2)-2,5-diphenyltetrazolium bromide (MTT) is reduced by metabolically active cells, by the action of dehydrogenase enzymes, generating NADH and NADPH. The resulting intracellular purple formazan is then solubilized and quantified by spectrophotometry. To assess cell viability after a 30-min exposure to different ZnCl_2_ concentrations, the Vybrant MTT Cell Proliferation Assay Kit was used according to the manufacturer’s protocol (Thermo Fisher Scientific). Briefly, MTT was added to each well at a final concentration of 0.15 mg/ml and incubated for 1 h at 37 °C. The dye was solubilized in DMSO, and the absorbance of each sample was measured at 570 nm in a 96-well plate spectrophotometer (Multiskan, Thermo Fisher Scientific). The data were normalized to the absorbance in the vehicle-treated cells (100%) and expressed as a percent of the control ± SEM from three separate experiments.

### Data Analysis

Results were obtained from at least three independent experiments. Data are shown as the mean ± standard deviation (SD). The experimental data were analyzed using GraphPad Prism 7 for Windows (GraphPad Software, San Diego California USA, www.graphpad.com).

Data from the saturation (*K*_D_), competition binding (*K*_i_), and kinetic experiments (k_off/offobs_) were fitted to a *one-* or *two-site* model, and the models were compared using an *F* test (extra sum-of-squares test).

The saturation binding data with respect to allosteric interactions was analyzed according to Eq. (1) [32]:

1 $$ {pK}_{\mathrm{App}}=- \log \left(\left[ B\right]+{10}^{\log {K}_B}\right)+ \log \left(\alpha \left[ B\right]+{10}^{\log {K}_B}\right)- \log d $$

where *K*_App_ is the apparent equilibrium dissociation constant for radioligand A in the presence of modulator B (ZnCl_2_); *K*_*A*_ and *K*_*B*_ are the equilibrium dissociation constants for the radioligand and allosteric modulator, respectively; log*d* is a constant representing the logarithm of the quotient of *K*_*A*_ and *α*; and *α* defines the cooperativity factor, which is the magnitude by which the equilibrium dissociation constant of either ligand for its site on the receptor is modified by the concomitant presence of the other ligand. Values of *α* less than 1 (but greater than zero) denote negative cooperativity, values greater than 1 denote positive cooperativity, and values that are not significantly different from 1 indicate neutral cooperativity.

For the competition binding assay, the affinity constant values (*K*_i_) were calculated using the Cheng–Prusoff equation (Eq. 2) [33]:

2 $$ {K}_i=\frac{IC_{50}}{1+\frac{L}{K_{\mathrm{D}}}} $$

where *IC*_50_ is the concentration of compound producing 50% inhibition of radioligand binding, *L* is the concentration of radioligand used, and *K*_D_ is the radioligand equilibrium dissociation constant that was obtained using the non-linear regression analysis from the saturation experiment.

The derivation of the Cheng–Prusoff equation was applied for the analysis of antagonist functional inhibition curves [34] (Eq. 3):

3 $$ {K}_B=\frac{IC_{50}}{1+\frac{A}{EC_{50}}} $$

where *K*_*B*_ is an antagonist dissociation constant, *A* is an agonist concentration, *IC*_50_ is the concentration of an antagonist producing a 50% reduction in the response to an agonist, and *EC*_50_ is the agonist concentration that causes half of the maximal response.

The dissociation kinetic data were fitted to the mono-exponential decay (Eq. 4):

4 $$ {B}_t={B}_0{e}^{-{k}_{\mathrm{offobs}}\bullet t} $$

where *B*_*t*_ denotes the radioactivity at time *t*, *B*_0_ denotes the radioactivity before the addition of zinc (time = 0), and *k*_offobs_ denotes the observed radioligand dissociation rate constant in the presence of the modulator. When the modulator is absent, *k*_offobs_ is the *k*_off_ for the radioligand.

The statistical significance of differences between means was evaluated using Student’s *t* test. The level of significance was established with *P* < 0.05.

## Results

The influence of zinc ions on the 5-HT_7_ receptor was evaluated in a series of radioligand binding and functional assays using recombinant HEK293 cells expressing the human 5-HT_7_ receptor. Since probe dependence is one of the hallmarks of allosteric protein regulation [1, 2], saturation, competition, and kinetic studies were performed with the following three different radiochemicals: the agonist [^3^H]5-CT and two antagonists that are selective ([^3^H]SB-269970) and non-selective ([^3^H]mesulergine) blockers of the 5-HT_7_ receptor. Both antagonists have also been described as inverse agonists of human 5-HT_7_ receptors overexpressed in HEK293 cells with high level of constitutive activity [35, 36].

**Fig. 1:**
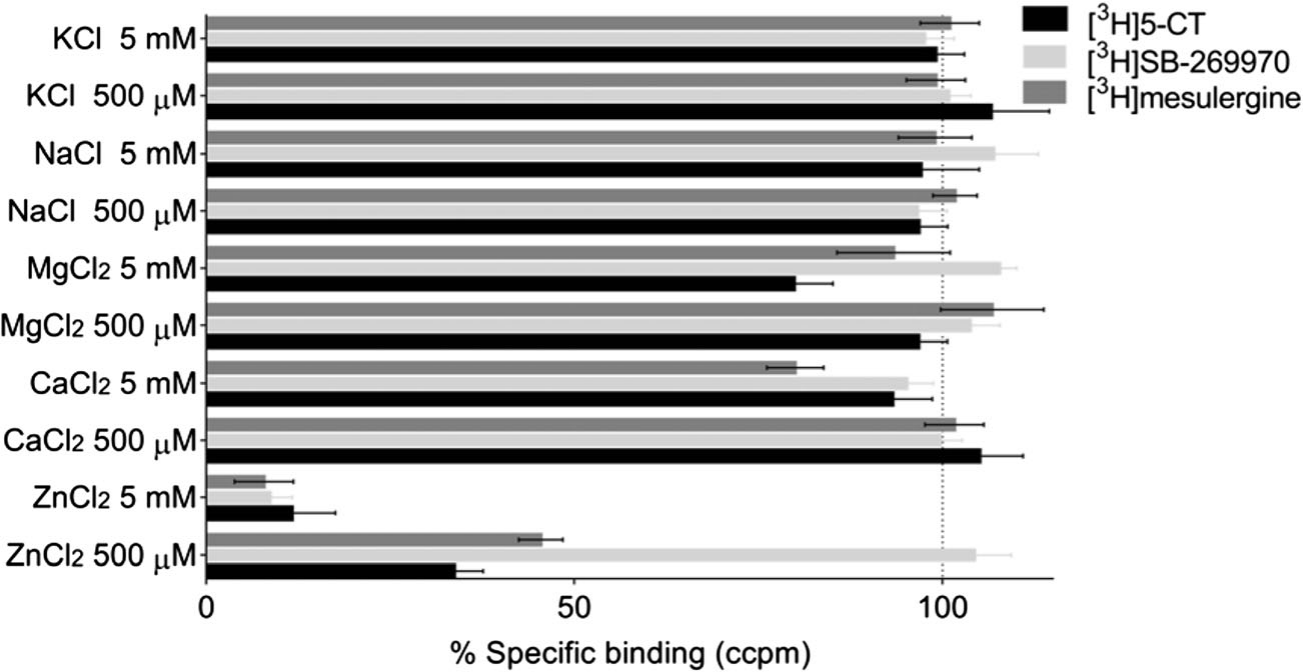
Effects of monovalent and divalent cations (Na^+^, K^+^, Mg^2+^, Ca^2+^, and Zn^2+^) at concentrations of 500 μM and 5 mM on the binding of [^3^H]5-CT, [^3^H]SB-269970, and [^3^H]mesulergine to human recombinant 5-HT_7_ receptors expressed in HEK293 cells. The binding of radioligands in the absence of the tested cations is referred to as basal specific binding and was set to 100% (*dotted line*)

Initially, different monovalent and divalent cations (Na^+^, K^+^, Mg^2+^, Ca^2+^, and Zn^2+^) were screened at concentrations of 500 μM and 5 mM to assess whether they had any effects on the 5-HT_7_ receptor (Fig. 1). Monovalent ions (Na^+^ and K^+^) at both concentrations and Ca^2+^ and Mg^2+^ ions at a concentration of 500 μM did not alter the binding of the three tested radioligands to the 5-HT_7_ receptor. Similarly, 500 μM zinc did not influence the level of [^3^H]SB-269970 binding, but it reduced [^3^H]5-CT and [^3^H]mesulergine binding by 67 and 55%, respectively (Fig. 1). Higher divalent ion concentrations have been shown to cause significant changes in the binding of the tested radioligands to 5-HT_7_ receptors. However, if the Mg^2+^ and Ca^2+^ ions decreased measurements by only approximately 10 to 20% relative to the control, Zn^2+^ caused changes in radioligand binding up to 90%. Therefore, the observed effects in the following radioligand binding experiments at the 5-HT_7_ receptor were assumed to be specific for zinc ions.

In saturation experiments performed in the absence of zinc, all radioligands bound to the 5-HT_7_ receptor in a monophasic manner and had Hill coefficients close to 1. The agonist [^3^H]5-CT labeled ca. 55–60% of the 5-HT_7_ receptors expressed in HEK293 cells (50.0 pmol/mg of protein) relative to both antagonists [^3^H]SB-269970 and [^3^H]mesulergine (81.1 and 91.2 pmol/mg of protein, respectively; Table 1).

**Table 1:** Effects of zinc on the binding parameters (*K*_D_ and B_max_) of [^3^H]5-CT, [^3^H]SB-269970, and [^3^H]mesulergine, obtained in saturation experiments of the 5-HT_7_ receptor expressed in HEK293 cells

Zn^2+^ [μM]	[^3^H]5-CT		[^3^H]SB-269970		[^3^H]mesulergine	
	*K*_D_ [nM]	B_max_ [pM/mg]	*K*_D_ [nM]	B_max_ [pM/mg]	*K*_D_ [nM]	B_max_ [pM/mg]
0	0.8 ± 0.1	50.0 ± 7.3	3.0 ± 0.2	81.1 ± 13.5	9.6 ± 0.8	91.2 ± 10.0
10	1.1* ± 0.1	52.5 ± 9.0	3.1 ± 0.4	78.8 ± 14.7	19.7* ± 0.1	76.4 ± 12.1
100	1.1* ± 0.1	55.1 ± 3.5	3.6 ± 0.6	89.2 ± 10.2	21.7* ± 1.6	72.2 ± 9.4
200	1.9* ± 0.1	57.7 ± 4.4	6.4* ± 0.3	93.7 ± 15.0	31.4* ± 1.5	116.7 ± 24.8
500	2.5* ± 0.2	60.6 ± 10.2	12.1* ± 0.6	124.3* ± 10.9	43.8* ± 2.2	105.6 ± 13.9
1000	5.9* ± 0.3	71.7* ± 7.2	18.1* ± 0.3	110.1* ± 7.1	58.3* ± 4.9	51.0* ± 3.9
2500	4.4* ± 0.2	43.0 ± 10.2	27.2* ± 1.4	107.4* ± 8.7	39.9* ± 0.5	39.0* ± 10.2
5000	4.3* ± 0.3	38.5* ± 2.5	28.7* ± 3.0	83.5 ± 16.2	34.6* ± 1.8	12.1* ± 2.6

*p < 0.05

This result was consistent with the findings of Alberts et al. [37], who showed that the agonist [^3^H]5-CT labeled 60% of the 5-HT_7_ receptors in comparison to the antagonist [^3^H]mesulergine and postulated the presence of two affinity states in the 5-HT_7_ receptor expressed in HEK293 cells. On the other hand, other data indicated similar B_max_ values obtained for the agonist [^3^H]5-CT, and the selective antagonist [^3^H]SB-269970 [38], as well as for [^3^H]mesulergine and [^3^H]5-CT binding [39].

In the present study, however, statistical analysis (GraphPad Prism 7) of the data from saturation experiments conducted in the absence of zinc ions did not reveal a preference for multi-site versus single-site interactions, and the obtained Hill coefficients ~1 indicated competitive binding of all radioligands to the 5-HT_7_ receptor. Based on that, the *K*_D_ values calculated for [^3^H]5-CT, [^3^H]SB-269970, and [^3^H]mesulergine were 0.8, 3.0, and 9.6 nM, respectively, which were slightly higher than those reported in the abovementioned publications (0.4, 1.4, and 6.2 nM, respectively) [37–39].

Because the main focus of our study was to investigate potential effects of zinc on 5-HT_7_ receptors, further saturation experiments were performed in the presence of seven increasing concentrations of Zn^2+^ ions (10 μM–5 mM).

**Fig. 2:**
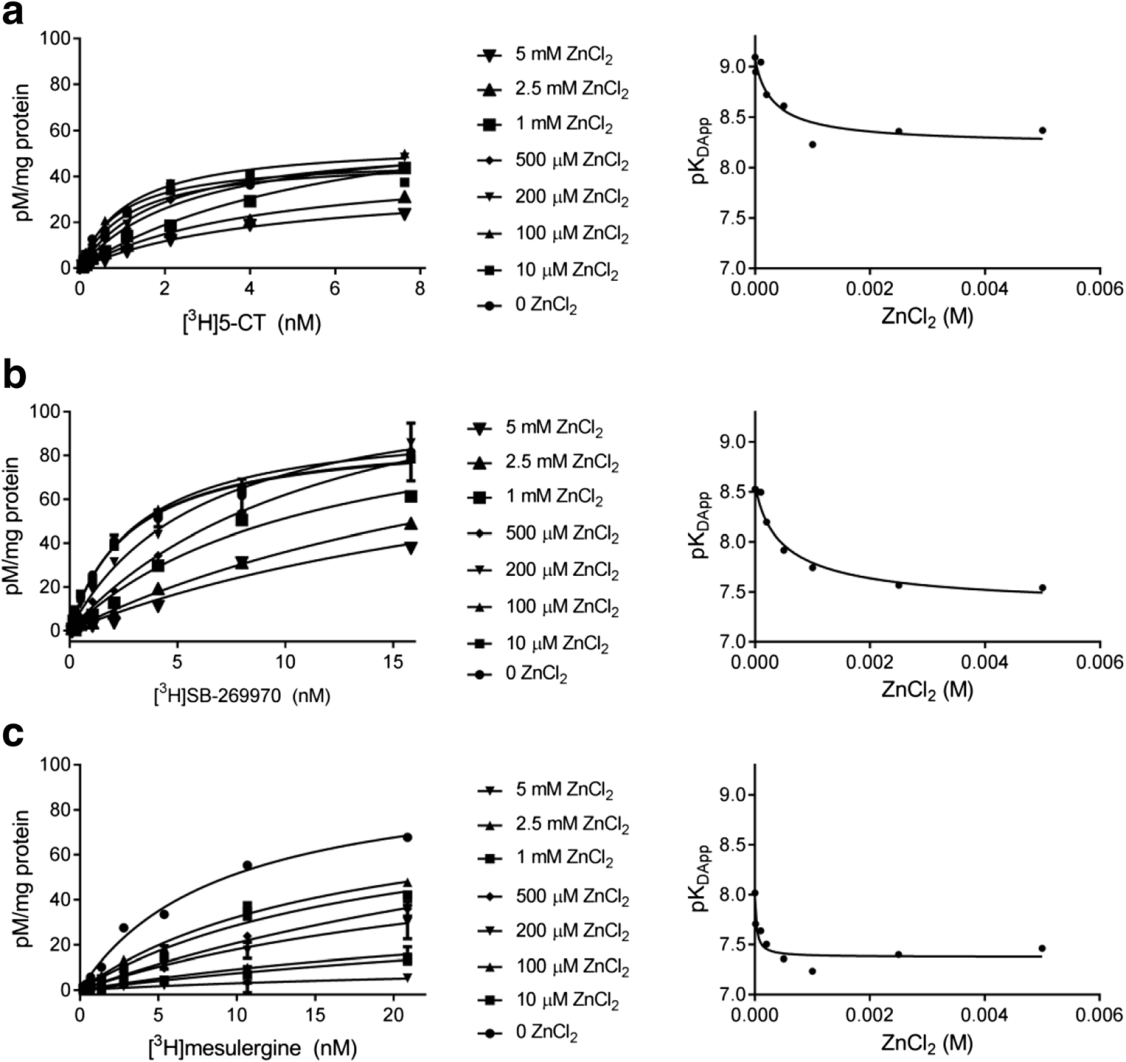
Effect of increasing concentrations of zinc ions on the binding of radioligands: **a** [^3^H]5-CT, **b** [^3^H]SB-269970, and **c** [^3^H]mesulergine to human recombinant 5-HT_7_ receptors expressed in HEK293 cells. The graphs in the *right panel* (which are diagnostic for negative allosteric modulation) show the non-linear regression analysis of the respective saturation data (*left panel*) according to Eq. (1)

As shown in Table 1 and Fig. 2, zinc caused an increase in the *K*_D_ values of all three radioligands, but the extent of the detected inhibitory effects varied. Regarding [^3^H]5-CT and [^3^H]mesulergine, statistically significant changes in *K*_D_ values were observed for all concentrations of Zn^2+^ (10 μM–5 mM), achieving a plateau at 1 mM of Zn^2+^. Such a ceiling effect (reaching a limit at high modulator concentrations) is considered to be a hallmark feature of allosterism [1]. For [^3^H]SB-269970, apparent changes in *K*_D_ were detected beginning at a concentration of 200 μM of Zn^2+^, and the maximum *K*_D_ was obtained at the two highest Zn^2+^ concentrations (Table 1).

Simultaneously, the number of binding sites for [^3^H]5-CT binding remained roughly unchanged, except for the concentration of 1 mM of Zn^2+^, for which a slight increase in [^3^H]5-CT binding capacity was noticed, and 5 mM of Zn^2+^, which was associated with significantly reduced B_max_ values. For [^3^H]SB-269970, an increase in B_max_ values was observed from 500 μM to 2.5 mM of Zn^2+^. For [^3^H]mesulergine, there was a statistically significant decrease in binding in the presence of high zinc concentrations (1–5 mM).

In general, changes observed in B_max_ values for all radioligands have a common tendency to increase and reach a maximum level at zinc concentrations in the range of 500 μM–1 mM. This is followed by a reduction of binding capacity, which for [^3^H]5-CT and [^3^H]mesulergine reached a significantly lower level than the control values, but in the case of [^3^H]SB-269970 it returned to the starting values. It should be noted, however, that the increased number of binding sites predicted for [^3^H]5-CT and [^3^H]SB-269970 might be overestimated, because they were determined based on extrapolation to very high radioligand concentrations (not used in our experimental procedure for practical reasons). Moreover, the observed increase in B_max_ values was not accompanied by an increase in radioligand affinity, as is the case of positive allosteric modulation. On the other hand, a decrease in the maximal number of binding sites probably results from allosteric inhibition causing changes in 5-HT_7_ receptor conformation, hindering the interactions of [^3^H]5-CT and [^3^H]mesulergine within the orthosteric binding pocket. These effects seem to be specific to zinc ions, because other monovalent and divalent cations at a concentration of 5 mM did not strongly inhibit binding of the investigated radioligands. Thus, ionic strength did not play an important role in non-specific ligand binding.

Importantly, a significant reduction in B_max_ values at millimolar concentrations of zinc has been observed for allosteric modulation of other GPCRs, as in the case of [^3^H]WAY200635 and [^3^H]8-OH-DPAT binding to 5-HT_1A_ receptors [7], interactions of dopamine D_1_ and D_2_ receptor selective antagonists [8], and non-competitive inhibition of antagonist binding to α_2_-adrenergic receptors [12].

The mechanism by which zinc inhibited the binding of the investigated radioligands in the saturation assays was analyzed by non-linear regression according to Eq. 1 [32]. The values obtained for the cooperativity factor *α*—0.15, 0.06, and 0.25 for [^3^H]5-CT, [^3^H]SB-269970, and [^3^H]mesulergine, respectively—together with the hyperbolic plots (Fig. 2) evidenced the negative allosteric modulation of Zn^2+^ ions at the 5-HT_7_ receptor.

It should be noted, however, that the *α* value approaching zero for Zn^2+^ cooperativity in [^3^H]SB-269970 binding denotes an interaction that is virtually indistinguishable from competition [1, 4].

In addition to saturation experiments, the influence of zinc on the 5-HT_7_ receptors was measured in competition assays. At first, Zn^2+^ titration curves against a single, fixed concentration (≤*K*_D_) of the investigated radioligand were evaluated, and the measured potency of Zn^2+^ for inhibiting the binding of [^3^H]5-CT was almost comparable to the potency obtained for [^3^H]mesulergine (IC_50_ values of 346 and 241 μM, respectively). Significantly weaker Zn^2+^ interactions were determined for [^3^H]SB-269970 binding, in which the IC_50_ value of Zn^2+^ was 1170 μM.

Subsequent competition experiments were performed for non-radioactive 5-CT, SB-269970, and clozapine (a non-selective 5-HT_7_ antagonist) versus the binding of [^3^H]5-CT, [^3^H]SB-269970, and [^3^H]mesulergine (used at a concentration ≤*K*_D_). The affinity values (*K*_i_) obtained in the absence and presence of two different concentrations of zinc ions (10 and 500 μM) are presented in Table 2.

**Table 2:** Effect of zinc ions at concentrations of 10 and 500 μM on 5-CT, SB-269970, and clozapine binding measured in competition experiments with [^3^H]5-CT (0.8 nM), [^3^H]SB-269970 (2.5 nM), and [^3^H]mesulergine (10 nM)

Zn^2+^ [μM]	[^3^H]5-CT			[^3^H]SB-269970			[^3^H]mesulergine		
	5-CT	Clozapine	SB-269970	5-CT	Clozapine	SB-269970	5-CT	Clozapine	SB-269970
0	0.76 ± 0.13	36.00 ± 1.41	2.82 ± 0.06	0.85 ± 0.21	23.55 ± 4.95	N.D.	0.79 ± 0.61	35.45 ± 6.36	N.D.
10	1.36* ± 0.08	32.50 ± 2.12	2.40 ± 0.06	1.05 ± 0.07	25.31 ± 12.73	N.D.	1.31 ± 0.64	43.18 ± 8.49	N.D.
500	3.76* ± 0.43	51.41* ± 7.18	4.86* ± 0.93	5.92* ± 0.07	92.56* ± 12.02	N.D.	13.21* ± 4.58	126.38* ± 32.14	N.D.

*N.D.* not determined

*p < 0.05

In the homologous agonist competition assay with [^3^H]5-CT, all the competition curves fitted a one-site binding model, either in the absence or the presence of zinc ions. A reduction of [^3^H]5-CT binding was visible at both Zn^2+^ concentrations, which was generally in agreement with the results of the saturation experiments. The addition of zinc ions also influenced the heterologous displacement of [^3^H]5-CT with clozapine and with SB-269970 from the 5-HT_7_ binding sites, but the inhibition of both antagonists was detected only at a concentration of 500 μM of Zn^2+^ and led to a 1.4- and 1.7-fold increase in their *K*_i_ values, respectively. Similarly, in the case of competition experiments with [^3^H]SB-269970, the affinity of 5-CT and clozapine decreased (6.9- and 3.9-fold, respectively) only at the higher zinc concentration (Table 2).

For [^3^H]mesulergine binding, both Zn^2+^ concentrations inhibited the 5-CT competition, but significant changes were detected in the presence of 500 μM of Zn^2+^. When [^3^H]mesulergine was displaced from the 5-HT_7_ binding sites by clozapine, the negative impact of Zn^2+^ was again noticeable only at higher Zn^2+^ concentration (Table 2).

For each heterologous ligand/radioligand competition curve, the one-site binding model provided the best fit, and single affinity constants were obtained in the absence and presence of zinc ions.

A fundamental method to confirm allosterism is to detect a change in the dissociation kinetics of an orthosteric ligand [1, 4]. Consistent with established knowledge that the kinetic effects of negative allosteric modulation should manifest at high rather than low concentrations of the modulator [40], and taking into account the observed inhibitory effects of zinc in competition experiments, the influence of Zn^2+^ on the kinetic performance of [^3^H]5-CT, [^3^H]SB-269970, and [^3^H]mesulergine was evaluated at a concentration of 500 μM (Table 3).

**Table 3:** Dissociation rate constants for [^3^H]5-CT, [^3^H]SB-269970, and [^3^H]mesulergine at the 5-HT_7_ receptor expressed in HEK293 cells, in the absence and presence of 500 μM of zinc ions

Zn^2+^ [μM]	Dissociation rate	[^3^H]5-CT	[^3^H]SB-269970	[^3^H]mesulergine
0	k_off_ [min^−1^]	0.070 ± 0.008	0.075 ± 0.008	0.059 ± 0.035
500	k_offobs_ [min^−1^]	0.092* ± 0.005	0.068 ± 0.003	0.100* ± 0.007

*p < 0.05

**Fig. 3:**
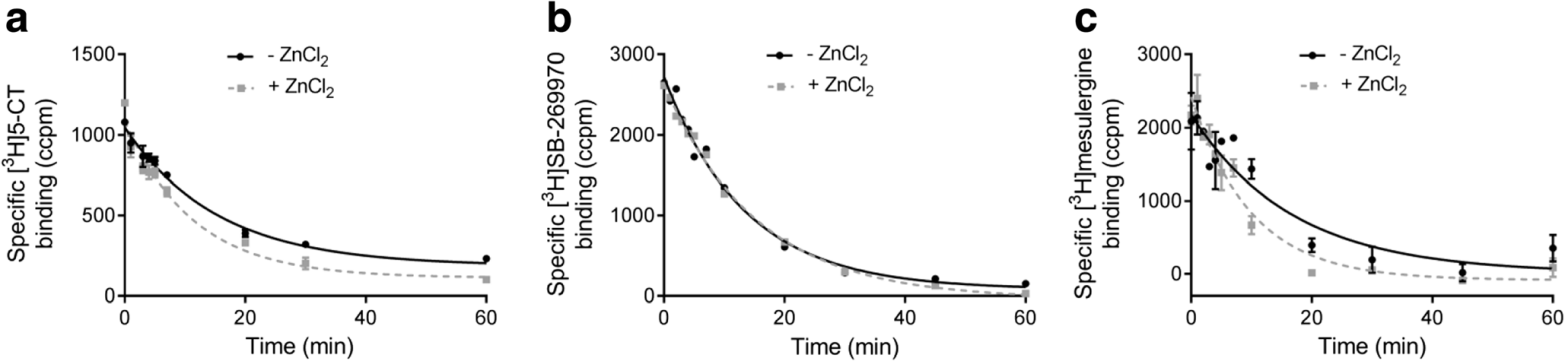
Effect of zinc (500 μM) on the dissociation rates of **a** [^3^H] 5-CT, **b** [^3^H]SB-269970, and **c** [^3^H]mesulergine from 5-HT_7_ receptors expressed in HEK293 cells. In all cases, the dissociation was induced by the addition of a receptor-saturating concentration of serotonin (10 μM)

In the absence of Zn^2+^, the dissociation rates of all investigated radioligands from 5-HT_7_ receptors, measured from 0 to 60 min at 37 °C, were monophasic and remained monophasic in the presence of zinc. The concentration of 500 μM of Zn^2+^ increased k_offobs_ values of [^3^H]5-CT and [^3^H]mesulergine but did not significantly affect the dissociation of [^3^H]SB-269970 from 5-HT_7_ receptors (Table 3 and Fig. 3).

**Fig. 4:**
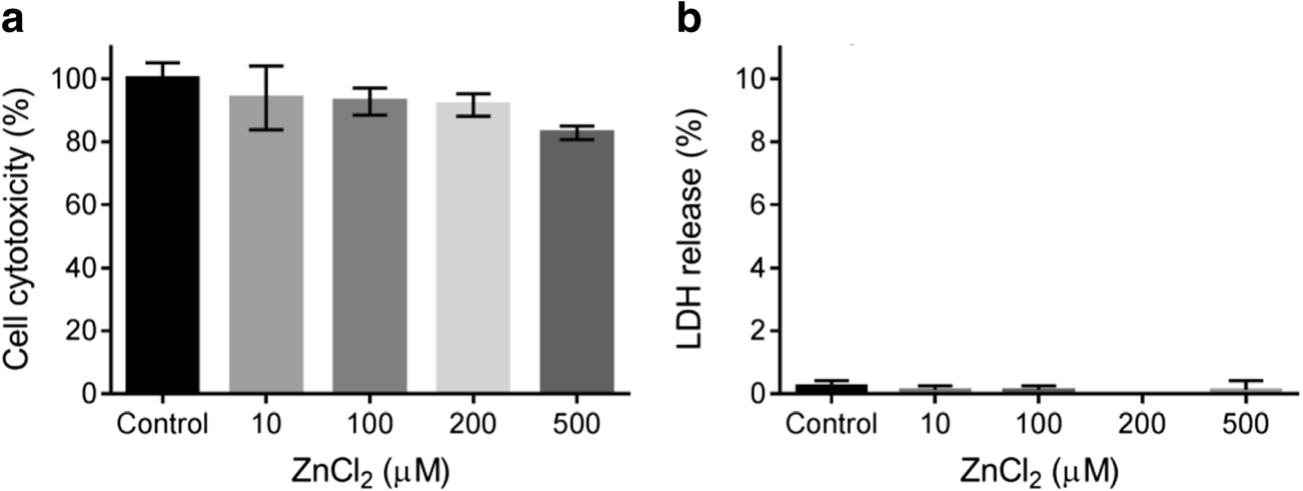
The cytotoxicity of zinc toward HEK293 cells was assessed in terms of **a** viability using the MTT assay and **b** mortality based on the release of lactate dehydrogenase (LDH). Cells were treated with different concentrations of zinc ranging from 10 to 500 μM. The results are presented as the means ± SD (*n* = 3), *p* < 0.05

**Fig. 5:**
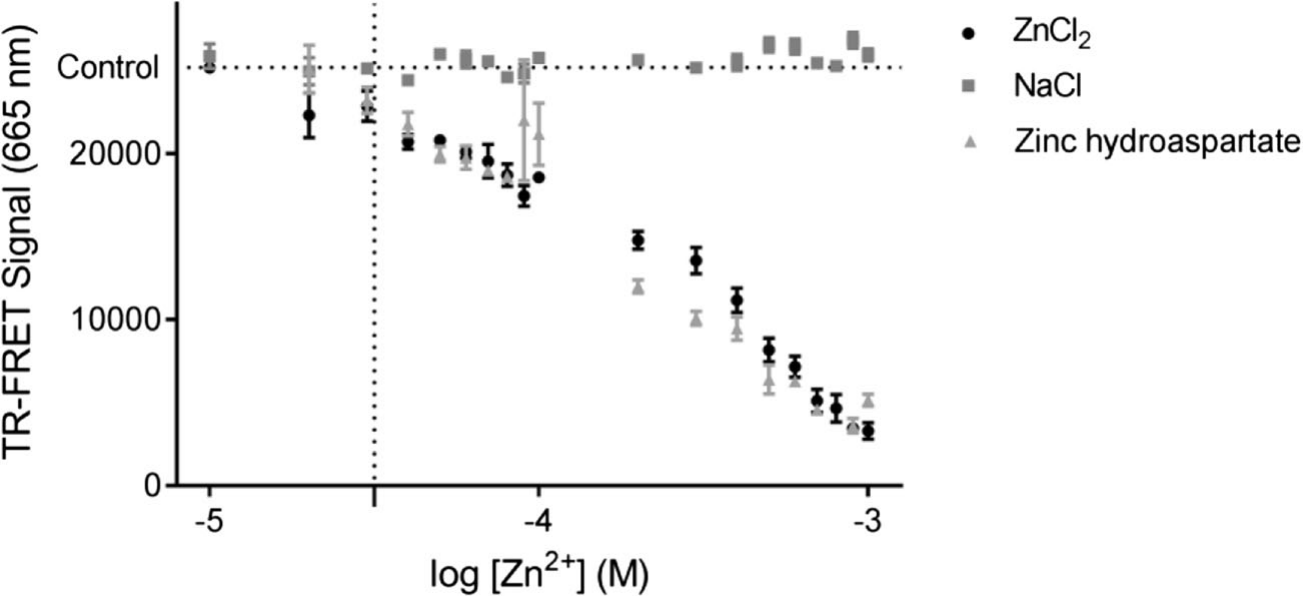
Influence of zinc salts (chloride or hydroaspartate form) and sodium chloride on PerkinElmer LANCE Ultra working solution Eu-cAMP tracer and U*Light*-anti-cAMP

Additional effects of zinc on 5-HT_7_ receptors were assessed in functional experiments using the cAMP accumulation assay in the same recombinant HEK293 cell line expressing the human 5-HT_7_ receptor. Initially, however, due to toxic effects of zinc that were detected in various cell systems, its influence on viability and membrane integrity in our cell line was investigated in MTT and LDH tests (Fig. 4a, b, respectively). Zinc ions up to a concentration of 200 μM did not affect the number of living cells. Additionally, because of their complexing properties, the potential interactions of Zn^2+^ ions with the components of the LANCE Ultra cAMP detection kit (PerkinElmer) used in our functional assays were also evaluated (Fig. 5). According to the PerkinElmer application note, heavy metal cations may quench fluorescence [41]. Indeed, Zn^2+^ ions at a concentration higher than 30 μM interacted with europium chelate (Eu-cAMP) and negatively influenced the fluorescence signal; a similar effect was observed for different zinc salts. Therefore, to avoid false-positive results, functional adenylyl cyclase activity was studied in the presence of up to 10 μM of Zn^2+^.

**Fig. 6:**
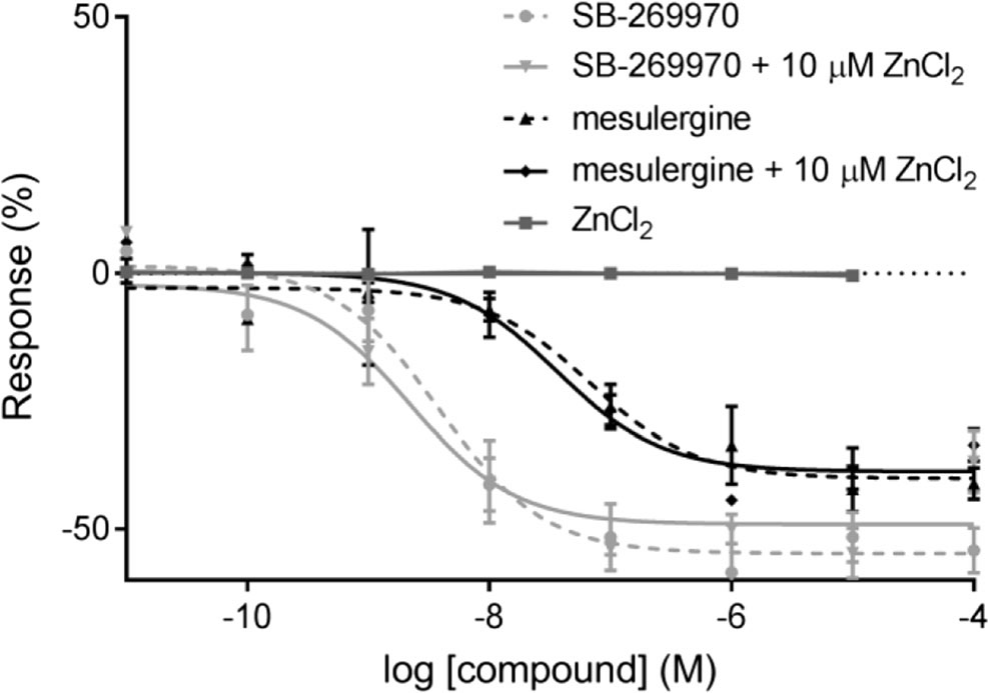
Effects of SB-269970 and mesulergine on unstimulated cAMP level in the 5-HT_7_ receptor expressed in HEK293 cells in the absence and presence of 10 μM of zinc ions, and zinc effects on spontaneous 5-HT_7_ receptor activity

A high level of constitutive activity present in our experimental system enabled visualization of the inverse agonistic properties of SB-269970 and mesulergine (Fig. 6). This result is in agreement with other studies describing the pharmacological profiles of these antagonists, in which human 5-HT_7_ receptors expressed in either HEK293 or CHO-K1 cells displayed constitutive activity [35, 42]. Because batches of HEK293 cells expressing 5-HT_7_ receptors used in separate experiments to measure inverse agonism showed some variation in absolute level of basal cAMP (35–50%), the effects of SB-269970 and mesulergine as inverse agonists were not quantified.

Besides, the overall pharmacological profiles of the investigated orthosteric ligands were consistent between batches. The 5-CT was a full agonist that stimulated cAMP production with values of EC_50_ = 0.84 nM and E_max_ = 100%, corresponding well to the binding affinity (*K*_i_ = 0.8 nM) determined in our radioligand studies. SB-26670 and mesulergine presented features of potent antagonists of the 5-HT_7_ receptor and inhibited 5-CT-induced cAMP accumulation with *K*_B_ values of 1.75 and 31 nM, respectively, and these results were similar to published data [42, 43].

**Fig. 7:**
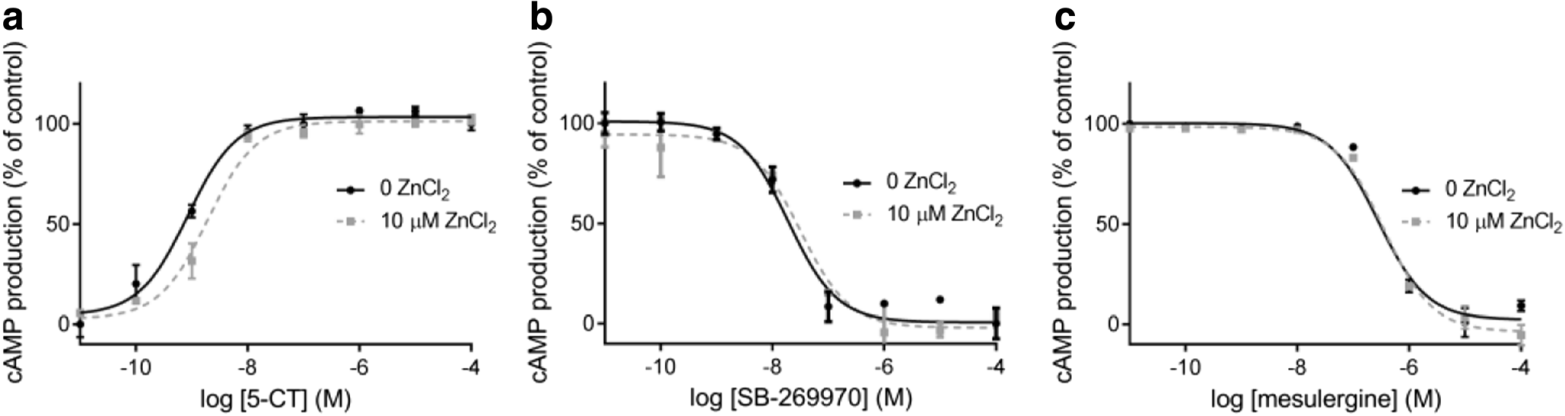
Concentration-response curves of **a** agonist 5-CT, **b** antagonist SB-269970, and **c** antagonist mesulergine, determined in the absence (*filled circle*) and presence of 10 μM (*grey square*) of zinc ions in recombinant 5-HT_7_ receptor-expressing HEK293 cells

Zinc alone had no influence on the level of constitutive activity, nor on the SB-269970 or mesulergine blockade of the spontaneous activity of the 5-HT_7_ HEK293 cell line (Fig. 6). However, zinc at a concentration of 10 μM produced a parallel rightward shift of the 5-CT dose-response curve, increasing the IC_50_ value by a factor of 3.2 (Table 4, Fig. 7a) with no significant diminution of the E_max_ value.

**Table 4:** Effects of zinc on the functional properties of 5-CT (EC_50_), SB-269970, and mesulergine (*K*_B_) at 5-HT_7_ receptor expressed in HEK293 cells

	Zn^2+^ [μM]	0	10
		*EC*_50_ [nM]	
Agonist	5-CT	0.84 ± 0.21	2.67* ± 0.32
		*K*_B_ [nM]	
Antagonist	SB-269970	1.75 ± 0.07	2.90* ± 0.06
	Mesulergine	31.05 ± 7.51	37.15 ± 6.86

*p < 0.05

Additionally, an influence of 10 μM of zinc on the effects of the functional blockade induced by SB-269970 on 5-CT-stimulated cAMP production via 5-HT_7_ receptors has been observed as a slight (but statistically significant) increase in the *K*_B_ values of this antagonist (by a factor of 1.6). In the parallel experiment, zinc did not alter the functional antagonism parameters for mesulergine (Table 4, Fig. 7b, c).

## Discussion

In recent years, various aspects related to targeting allosteric sites on GPCRs have been extensively studied, and most reports have concerned metabotropic glutamate receptors of class C GPCRs [44, 45]. In rhodopsin-like (or class A) receptors, which are the largest class of GPCRs with 285 members, the phenomenon of allosteric modulation has been studied predominantly in adenosine and muscarinic receptors [1, 46–49].

Regarding the serotonergic GPCR family, there are only a few examples of modulatory regulation involving the action of oleamide on 5-HT_2A_ and 5-HT_7_ receptors [50, 51], positive modulation of PNU-69176E on the 5-HT_2C_ receptor [52] and cholesterol [53], and zinc allosteric modulation of the binding properties of the 5-HT_1A_ receptor [6, 7].

On the other hand, zinc, as an essential trace element in living organisms, plays many roles, including participating in multiple processes within the CNS [54]. In the brain, Zn^2+^ has been identified as a ligand that is capable of activating and inhibiting receptors, including NMDA-type glutamate receptors, GABA_A_ receptors, nicotinic acetylcholine receptors, glycine receptors, and ionotropic serotonin 5-HT_3_ receptors [24, 55]. The role of zinc in depression and antidepressant therapy was emphasized in numerous preclinical and clinical studies, but the precise mechanism of this action is not understood [28–30, 56–59]. It has been suggested that the effects of zinc can also be mediated by the serotonergic system [28, 60–62], among others, and the presence of endogenous Zn^2+^ binding sites in 5-HT_1A_ receptors, together with its allosteric regulation, has been demonstrated [6, 7].

The presented data indicate that the effects of Zn^2+^ on serotonin GPCRs are not restricted only to the modulation of the 5-HT_1A_ receptor subtype. Both radioligand and functional studies conducted using human 5-HT_7_ receptors stably expressed in the HEK293 cell line have shown that zinc also has the potential to regulate the activity of 5-HT_7_ receptors.

First, in the saturation assays performed in the absence and presence of increasing concentrations of zinc, changes in affinity were observed for all radioligands, i.e., the agonist [^3^H]5-CT and the antagonists [^3^H]SB-269970 and [^3^H]mesulergine. The obtained results demonstrated that zinc inhibits agonist and antagonist binding in a negatively cooperative manner; however, depending on the nature of the orthosteric ligand used, differences in the magnitude of the effect manifested in different values of cooperativity. In the case of [^3^H]5-CT and [^3^H]mesulergine binding, the calculated *α* values (0.15 and 0.25, respectively) indicate a negative allosteric mode of action, while the cooperativity factor calculated for [^3^H]SB-69970, which approaches zero (0.06), may also mean competitive interaction [1, 4]. The dissociation kinetic experiments further supported these conclusions. The accelerated dissociation rates for [^3^H]5-CT and [^3^H]mesulergine binding at 5-HT_7_ receptors measured in the presence of 500 μM of zinc are consistent with the negative allosteric modulation in which the effects on the orthosteric ligand affinity are generally mediated via enhanced dissociation [4].

On the other hand, the lack of substantial effects of Zn^2+^ on the [^3^H]SB-269970 dissociation rate implies that alterations in the affinity observed in the saturation experiments (associated with a small *α* = 0.06 value) resembled competitive antagonism. Since allosterism is probe dependent, the changes detected for one radioligand may not be detected for another [1]. Numerous examples of probe dependence have been described for allosteric modulators with different degrees of cooperativity. For example, in the group of the most frequently investigated muscarinic acetylcholine receptors, the prototypical allosteric modulator, alcuronium, produced a 10-fold change in the affinity of acetylcholine for the muscarinic M2 receptor but only a 1.7-fold change in the affinity of another muscarinic cholinergic agonist, arecoline [1]. On the other hand, regarding the displacement of the antagonist [^3^H]methyl-QNB, alcuronium reduced radioligand binding to a non-specific level, while it enhanced the binding of [^3^H]atropine [63].

The observed differences in the effects of zinc on orthosteric radioligand interactions at 5-HT_7_ receptors were also detected in the competition-like titration experiments, in which the IC_50_ values obtained for zinc ions indicated three to four times more potent inhibition of [^3^H]5-CT and [^3^H]mesulergine than [^3^H]SB-269970 binding. In the cross-competition experiments using non-labeled orthosteric ligands (5-CT, SB-269970, and clozapine) and a fixed concentration (~*K*_D_) of a given radioligand, the presence of 500 μM of zinc ions caused a moderate but statistically significant decrease in non-labeled orthosteric ligand affinities, which again confirmed the inhibitory effects of Zn^2+^ on the orthosteric binding at 5-HT_7_ receptors. In addition to the radioligand binding studies, the influence of Zn^2+^ on 5-HT_7_ receptor signaling has also been evaluated in a functional LANCE test. It has been reported that zinc can have diverse signaling effects on different GPCRs, behaving as an agonist, as an allosteric modulator, or as an inverse agonist [11–15, 64, 65]. Therefore, the signaling effects of Zn^2+^ were examined in our cell line, which exhibited a sufficiently high level of constitutive activity needed to distinguish neutral (silent) antagonists and inverse agonists. By preferentially binding to and stabilizing the inactive receptor state, inverse agonists reduce spontaneous receptor activity. However, neutral antagonists display equal preferences for both inactive and active states and have no intrinsic activity. They are able to block the actions produced by either agonists or inverse agonists [66].

In the presently conducted functional tests using the constitutively active 5-HT_7_ HEK293 cell line, a zinc concentration up to 10 μM (which does not produce false-positive signals due to interactions with the LANCE reagents) behaved as a weak neutral antagonist. Zinc did not change the level of spontaneous receptor activity, and it blocked 5-CT-induced cAMP production. Zinc had no influence on the blockade of spontaneous activity by the inverse agonists SB-269970 and mesulergine; however, it weakened the functional antagonism (i.e., the ability to block the agonist-mediated response) of SB-269970.

Taken together, these results demonstrated that apart from the negative allosteric modulation of orthosteric 5-HT_7_ receptor ligand binding, zinc also inhibited the signaling properties of the 5-HT_7_ receptor. These effects can be included in the pleiotropic role of zinc in the CNS, as documented by numerous studies investigating its potential to act as an allosteric modulator of various signaling systems. Indeed, there are many examples of the positive or negative influence of extracellularly applied zinc on orthosteric ligand binding and function at several GPCRs, such as dopamine, adrenergic, melanocortin, and opioid receptors [5, 8–18].

As already mentioned, our previous studies on zinc allosteric regulation of serotonin 5-HT_1A_ receptor indicated that in a case of agonist binding, biphasic effects involving the potentiation at Zn^2+^ sub-micromolar concentrations (10 μM) and the inhibition at Zn^2+^ sub-millimolar concentrations (500 μM) were found in radioligand binding assays, which seems to be consistent with both the agonist and the antagonist-like effects of zinc ions observed in in vivo studies [7]. Earlier investigations have also shown negative allosteric properties of zinc against 5-HT_1A_ receptor antagonist binding [6].

Here, we showed new data obtained using radioligand binding studies regarding the allosteric inhibition by zinc of both agonist and antagonist interactions with 5-HT_7_ receptors together with neutral antagonism by a zinc ion concentration of 10 μM in the functional assay. Since the 5-HT_7_ receptor is coupled to the Gs protein and the receptor stimulation results in the activation of adenylyl cyclase leading to an increase in cAMP production [67], its blockade by zinc ions will decrease the concentration of cAMP. On the other hand, stimulation of the 5-HT_1A_ receptor, which is known to activate a variety of effectors via Gi/o proteins, leads to the inhibition of adenylyl cyclase [68–70]. Therefore, as found in our previous study, the potentiation of agonist binding to 5-HT_1A_ receptors in the presence of 10 μM of zinc ions should reduce the cAMP concentration (confirmed in our recent functional assays with the HEK293 cells stably expressing the human 5-HT_1A_ receptor; data not shown). Given that the reported range of extracellular free Zn^2+^ concentrations is below 100 μM [71], the effects observed with 10 μM of zinc represent the most likely direction of zinc action under physiological conditions, and similarly to the 5-HT_1A_ receptor, the 5-HT_7_ receptor can also be considered a serotonergic target for zinc modulation in the CNS. Taken together, although the 5-HT_1A_ and 5-HT_7_ receptors differ in terms of intracellular signaling, the functional consequences of zinc ions at a concentration of 10 μM at both receptors should result in the same decreasing effect on cAMP production and on further neuronal excitability. This is consistent with the antidepressant properties revealed by either the agonists of the 5-HT_1A_ receptor and the antagonists of the 5-HT_7_ receptor in various behavioral models [72–77]. Moreover, because the 5-HT_1A_ and 5-HT_7_ receptors are co-expressed in various brain regions and their functional cross talk has been suggested in depressive disorders, possibly through heterodimerization [25, 78], the fine-tuning of receptor-mediated signaling by zinc ions can be hypothesized. It has also been recently postulated that zinc plays a critical role in specific interactions between 5-HT_1A_ receptor and galanin receptor 1 by disrupting formation of pathological heterodimers in a depressive state [61, 62]. Therefore, in future studies it would be interesting to analyze the involvement of zinc ions also in the process of 5-HT_1A_/5-HT_7_ receptor heterodimerization.

In summary, the results of the present study showed that zinc can act as a negative allosteric modulator of the 5-HT_7_ receptor. According to our knowledge, this is the second example of a 5-HT_7_ receptor allosteric modulation, which has been previously described only for oleic acid and its derivative oleamide [37, 50, 51]. The confirmation of the existence of an allosteric binding site on 5-HT_7_ receptors may lead to entirely new research areas with the potential to design exogenous small-molecule ligands based on the allosteric regulation of the 5-HT_7_ receptor. Moreover, in light of the neuromodulatory role of zinc, our results also support its additional function in the regulation of CNS homeostasis, which may partially explain the effectiveness of zinc supplementation in the treatment of depression through the allosteric modulation of both 5-HT_1A_ and 5-HT_7_ receptors.
